# Self-Reported Prevalence of Symptomatic Adverse Reactions to Gluten and Adherence to Gluten-Free Diet in an Adult Mexican Population

**DOI:** 10.3390/nu7075267

**Published:** 2015-07-21

**Authors:** Noe Ontiveros, Jesús A. López-Gallardo, Marcela J. Vergara-Jiménez, Francisco Cabrera-Chávez

**Affiliations:** 1 Regional Program for PhD in Biotechnology, FCQB, University of Sinaloa, Culiacán, Sinaloa 80019, México; E-Mail: noeontiveros@gmail.com; 2 Nutrition Sciences Academic Unit, University of Sinaloa, Culiacán, Sinaloa 80019, México; E-Mails: aristeo.lopez37@hotmail.com (J.A.L.-G.); mjvergara@uas.edu.mx (M.J.V.-J.)

**Keywords:** prevalence, adverse reactions, gluten-free diet, gluten-related disorders

## Abstract

The prevalence of symptomatic adverse reactions to gluten and adherence to gluten-free diet in Latin American countries is unknown. These measurements are strongly linked to gluten-related disorders. This work aimed to estimate the prevalence of adverse reactions to oral gluten and the adherence to gluten-free diet in the adult Mexican population. To reach this aim, a self-administered questionnaire was designed and tested for clarity/comprehension and reproducibility. Then, a self-administered questionnaire-based cross-sectional study was conducted in the Mexican population. The estimated prevalence rates were (95% CI): 11.9% (9.9–13.5) and 7.8 (6.4–9.4) for adverse and recurrent adverse reactions to gluten respectively; adherence to gluten-free diet 3.7% (2.7–4.8), wheat allergy 0.72% (0.38–1.37); celiac disease 0.08% (0.01–0.45), and NCGS 0.97% (0.55–1.68). Estimated pooled prevalence of self-reported physician-diagnosis of gluten-related disorders was 0.88% (0.49–1.5), and 93.3% respondents reported adherence to gluten-free diet without a physician-diagnosis of gluten-related disorders. Symptom comparisons between those who reported recurrent adverse reactions to gluten and other foods showed statistically significant differences for bloating, constipation, and tiredness (*p* < 0.05). Gluten-related disorders may be underdiagnosed in the Mexican population and most people adhering to a gluten-free diet are doing it without proper diagnostic work-up of these disorders, and probably without medical/dietician advice.

## 1. Introduction

Adverse reactions to foods can be mediated or not by immune mechanisms. Particularly, gluten related disorders such as wheat allergy and celiac disease are well known immune mediated conditions [1]. Another gluten related disorder is Non-Celiac gluten sensitivity (NCGS). These are cases where symptomatic adverse reactions to oral “gluten” occur, but in which neither allergic nor autoimmune mechanisms are involved [1,2]. However, other immune mechanisms related to the innate immune system could play a role in this condition, but the precise mechanisms underlying NCGS are not well understood yet [1,3]. Notably, all NCGS cases are symptomatic the hallmarks of this condition being gastrointestinal ornon-intestinal symptoms [4].

The existence of NCGS was supported a few years ago [5], but further studies carried out by the same group showed no gluten-specific symptomatic adverse reactions to gluten in a specific population [6]. This has led to the idea that other wheat components besides gluten could trigger the symptoms seen in NCGS cases. However, due to the lack of biomarkers for the diagnostic work-out of NCGS, a proper diagnosis of NCGS can be performed only after wheat allergy and celiac disease have been ruled-out, symptomatic relief is reached after gluten withdrawal, and gluten-specific symptoms are confirmed by a food challenge test [7,8].

Following a gluten-free diet is the only accepted treatment for those affected with celiac disease or NCGS. Wheat allergic patients do not necessary need to follow a gluten-free diet, but they have to avoid wheat in their diets. Although a gluten-free diet is costly and socially restrictive, it is possible that more people than those that truly benefit from it avoid gluten in their diets [9,10]. This could have some implications as adhering to a gluten-free diet could impact on the serum levels of some micronutrients such as iron and vitamin B [11,12]. Moreover, due to the reliability of celiac disease specific serology tests and pathology results are strongly influenced by gluten intake, adhering to a gluten-free diet without a proper diagnosis of celiac disease could complicate the diagnostic work-up of this condition [8].

Until now, it is been accepted that the prevalence of celiac disease is around 1% in the general population, but prevalence data about wheat allergy in adult population are scarce and data about NCGS highly differ. In fact, NCGS prevalence range from 1% to 13% according to studies carried out in the United States and Europe [1,13,14]. Furthermore, considering adherence to gluten-free diet in non-celiac population as a surrogate of NCGS, the prevalence of the latter condition in the United States is 0.55% [15], which is slightly lower than that of celiac disease (0.71%) [9]. Notably, although there are some prevalence data about celiac disease in Latin America, there is neither NCGS nor gluten-free diet adherence prevalence data in open populations within Latin American countries. Moreover, prevalence data related to other adverse reactions to foods such as food allergy are scarce. Identifying those cases that potentially develop symptomatic adverse reactions to foods is the first step to estimate with objective diagnostic criteria the prevalence of NCGS or other symptomatic adverse reactions to foods in population-based studies.

The main aim of this study was to estimate the prevalence of symptomatic adverse reactions to oral gluten and other foods as well as the adherence to gluten-free diet in the Mexican population aged 18 years or older. Moreover, due to the lack of a validated instrument to identify potential NCGS cases in a Latin population, an additional aim was to design and test a self-administered questionnaire to identify potential cases of symptomatic gluten-related disorders and other cases of adverse reactions to foods.

## 2. Experimental Section

### 2.1. Questionnaire Design

Based on two previously devised instruments and recognized NCGS symptoms published in scientific literature, a self-administered questionnaire was designed to identify people developing symptomatic adverse reactions to oral gluten and other foods. Gastrointestinal and extraintestinal symptoms were first recapitulated from an NCGS questionnaire devised by the Italian Celiac Disease Association and the Italian Celiac Foundation [14]. To ensure that most relevant NCGS-associated symptoms were included, additional scientific sources evaluating gluten-induced symptoms by oral gluten challenge test in NCGS population were consulted [5,16,17,18]. Thus, the NCGS-associated symptoms assessed in this study can be defined as literature-based NCGS symptoms and were chosen based on the frequencies of gastrointestinal and extraintestinal manifestations reported (material S3, questions 5 and 9).

**Figure 1:**
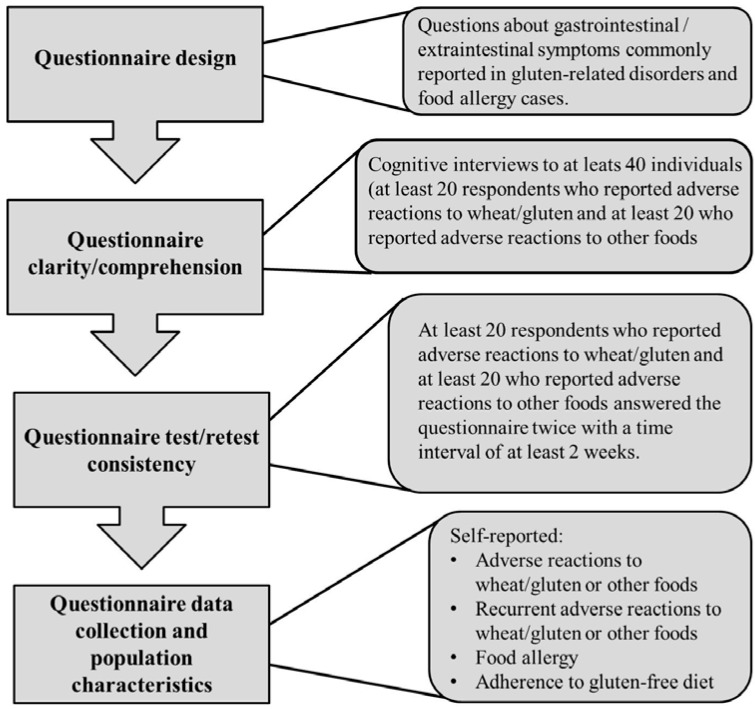
Flowchart of the study protocol.

Since the questionnaire devised by the Italian Celiac Disease Association/Foundation was designed for collecting clinical data by skilled investigators attending outpatient clinics, we took advantage of a previously validated Spanish version of an in-depth questionnaire intended to evaluate the prevalence of self-reported food allergy [19]. This instrument asks questions about typical symptoms of immediate hypersensitivity allergic reactions that have high sensitivity for positive specific food IgE [20,21]. Thus, the designed questionnaire includes not only items/questions related to NCGS, but also items/questions related to food allergy (Figure 1).

As expected, some items/questions of the in-depth Spanish questionnaire were adapted to ask questions about symptoms triggered by oral gluten or other foods, and to be self-administered. In this process a few words were changed from the original Chilean source to better fit the words for Mexican culture, but the ways to measure the variables of interest were not modified. To certify that the questions and responses were simple, clear, natural sounding, and the language used was at the level of understanding of the target population, we performed tests to evaluate comprehension, reproducibility, and wording of questions of the designed instrument.

### 2.2. Questionnaire Clarity/Comprehension and Wording of Questions Evaluation

Clarity/comprehension was evaluated as previously described [22]. This evaluation was performed by means of cognitive interviews in a sample of the target population (at least 20 subjects developing adverse reactions to gluten and 20 developing adverse reactions to other foods). The interviewees should evaluate each item/question in a three point scale; 1: Clear and comprehensible, 2: Difficult to understand, and 3: Incomprehensible (ordinal scale). Clarity/comprehension was also evaluated in a numerical scale from 0 to 10. For this evaluation 0 was considered very easy to understand and 10 very difficult to understand. The interviewees had to answer the question; how would you rate this item/question? Items/questions rated ≤3 were considered clear and comprehensible and, consequently, rewording was not required. We made sure that all possible answers were read/written immediately after the questions and the language used at the level of the target population. This can be helpful in cases of non-self-explanatory questions [23]. To verify the comprehension of the items and to evaluate the wording of questions, the interviewees should state with their own words the perceived meaning of each item and to answer the question: how would you write this item? These participants were identified in previous screening tests and were not included in the prevalence study.

### 2.3. Questionnaire Test-Retest Consistency

At least 20 subjects who reported adverse reactions to gluten and another 20 who reported adverse reactions to other foods answered the questionnaire twice. The time period interval between first and second application of the questionnaire was of at least two weeks (range: 2–4 weeks). These participants were identified in previous screening tests and were not included in the prevalence study. Intraclass or Lin’s concordance correlation coefficients are commonly used for measuring test-retest consistency. These methods are useful to assess agreement in continuous variables, but most variables to be measured with the designed instrument were categorical. Thus, test-retest consistency analysis using the Kappa statistics was performed and results with 95% confidence intervals were reported. In addition, consistency was also reported as percentage of matched questions between first and second application of the questionnaire. A matched question was considered when the respondents reported the same gastrointestinal or extraintestinal symptoms in the first and second application of the questionnaire. The same approach was applied for other questions where more than one answer could be chosen. For the analysis, first and second applications of the questionnaire were considered as rater one and two respectively.

### 2.4. Population Survey

The survey was conducted outside mainstream shopping malls and urban parks of Culiacan, Sinaloa, Mexico. All data were collected during the period from January to April 2015. Inclusion criteria were as follows: subjects aged 18 years or older and able to read and answer the questionnaire by themselves. Exclusion criteria included subjects less than 18 years old or subjects that were not able to complete the questionnaire by themselves. Trained nutritional science students gave assistance on specific terms when it was requested. The first part of the questionnaire asked about demographics information including age, gender, and education. This part also includes contact information and a key question about adverse reactions to oral gluten (do you have some discomfort or adverse reaction when consuming wheat products?). Race/ethnicity was not considered as the city of Culiacan, Sinaloa is not characterized for being multicultural. The first section included 15 items/questions and was designed for those who reported adverse reactions to oral gluten. The first question in this section asked about the diagnostic work-up of gluten related disorders (have you ever been diagnosed with a disease related to the consumption of wheat or gluten?). The second section includes 14 items/questions and was designed for those who reported adverse reactions to other foods different from gluten. This section enables the estimation of general self-reported prevalence of adverse reactions to foods and, consequently, to estimate the magnitude of the symptomatic adverse reactions to gluten in the studied population. Additionally, prevalence rates of adverse reactions to individual foods including self-reported food allergy prevalence rates could be estimated (supplemental material). First and second section screened for gastrointestinal and extraintestinal symptoms, symptom frequency, time interval between the ingestion of food and symptom occurrence, and family history of celiac disease. In addition, first section screened for gluten-related disorders and the person who performed the diagnosis. All participants answered the question; do you keep a diet free of wheat or gluten?

A reported diagnosis of celiac disease was considered when the respondents were diagnosed by a physician or and were also following a gluten-free diet [13]. In addition to the previous criteria, self-reported wheat/food allergy was also considered when the respondents reported recurrent adverse reactions “convincing” of food allergy (Figure 1). This includes skin with hives and angio-edema, trouble breathing, wheezing or throat tightness, vomiting, and diarrhea, and the symptoms occurred within 2 h after food ingestion [19,20,21], or the respondents reported a previous diagnosis of food allergy (supplemental material S3, questions 13 or 26). Recurrent adverse reactions were considered when the respondents reported that the food-induced symptoms occurred always or most of the times.

### 2.5. Statistical and Ethical Issues

Statistical analysis was carried out using PASW statistics version 18.0 (SPSS Inc., Chicago, IL, USA). Categorical variables were summarized by descriptive statistics, including total numbers, percentages, odds ratio, 95% confidence interval, and associations were analyzed by two-tailed Fisher exact test. Odds ratio analysis was conducted to identify the symptoms preferentially associated to recurrent adverse reactions to gluten compared to other foods. Respondents that reported other allergies different from wheat allergy or dairy intolerance, but also reported recurrent adverse reactions to gluten were included in the analysis. Exposed and non-exposed cases were represented by those who reported and non-reported a specific symptom respectively, and cases and controls were represented by those who reported recurrent adverse reactions to gluten and recurrent adverse reactions to other foods respectively. Continuous variables were summarized by mean and range with differences between two groups calculated using the Student *t*-test. A *p*-value < 0.05 was considered statistically significant. Prevalence rates were calculated using OpenEpi software version 3.03 [24]. Rates were reported as rate (95% confidence intervals) per 100 inhabitants. Questionnaire completion and return was regarded as consent. An Ethics Review Board of the Autonomous University of Sinaloa approved the study protocol (ethic approval number CE-UACNYG-2014-AGO-001).

## 3. Results

### 3.1. Questionnaire Evaluation

Clarity/comprehension was evaluated in a population that reported recurrent-food-induced symptoms. For this purpose, twenty-two respondents had to answer Section 1 of the instrument designed (adverse reactions to gluten). Among these, eight respondents reported gluten-induced gastrointestinal and extraintestinal symptoms (36.4%). For Section 2 (adverse reactions to other foods), eight out of 20 respondents reported gastrointestinal and extraintestinal adverse reactions to foods different from gluten (40.0%). The school degree of the respondents in each section of the instrument ranged from elementary to postgraduate. Overall, clarity/comprehension evaluation was excellent with an ordinal scale score of clear and comprehensible, and numerical score of zero which means very easy to understand (Table 1). In line with this, when the respondents were asked a key question to verify comprehension and wording of items/questions (how would you write this item/question?), all of them answered that they would leave the items/questions as they were originally written. Consistency was evaluated in a separate group of respondents. This group shared characteristics with the clarity/comprehension group (as described above). Eight out of 20 respondents in Section 1 (40.0%) and 11 out of 24 in Section 2 (45.8%) reported a combination of recurrent-food-induced gastrointestinal and extraintestinal symptoms respectively. Percentages of matched questions between first and second application of the instrument were of 92.7% (range: 50%–100%) and 78.8% (range: 58.3%–100%) for Section 1 and Section 2 respectively. The measure of agreement Kappa was found to be 0.92 (*p* < 0.001) and 0.77 (*p* < 0.001) for Section 1 and Section 2 respectively (Table 1). These Kappa values can be interpreted as almost perfect agreement and moderate agreement respectively [25].

**Table 1:** Study population and test results of the designed questionnaire.

Assessment	Age Mean (Range)	*n* (Female/Male)	Adverse Reactions	Score
Clarity/comprehension	34.9 (20–66)	22 (5/17)	Gluten	Clear and comprehensible (0) *
	29.5 (18–50)	20 (12/8)	Other foods	Clear and comprehensible (0)
Consistency	33.2 (20–63)	20 (12/8)	Gluten	0.918 ^#^ (0.88–0.96) ^##^
	22.1 (20–43)	24 (19/5)	Other foods	0.767 (0.71–0.82)

* Score in numeric scale. The Spanish version of the questionnaire was evaluated; ^#^ Measure of agreement Kappa; ^##^ 95% confidence interval.

### 3.2. Population Survey

A total of 1238 participants answered and returned the questionnaire (54.85% were female and 45.15% were male). The general prevalence of self-reported gluten-related disorders was 1.77% (*n* = 22) (95% CI: 1.2–2.7). Prevalence rates of self-reported NCGS, celiac disease, and wheat allergy were 0.97% (95% CI: 0.55–1.68), 0.08% (95% CI: 0.01–0.45), and 0.72% (95% CI: 0.38–1.37) respectively. The characteristics of this population are given in Table 2. The prevalence rate of self-reported physician-diagnosed NCGS was 0.81% (95% CI: 0.41–1.53). Notably, only 27.3% (*n* = 6) of the self-reported gluten-related disorders group (*n* = 22) was following a gluten-free diet (Table 2). Overall, the prevalence rate of self-reported gluten-related disorders currently following a gluten-free diet was 0.48% (95% CI: 0.22–1.05).

**Table 2:** Characteristics of identified self-reported gluten-related disorder cases.

Gluten-Related Disorder	Mean Age in Years (Range)	Number of Cases (Female/Male)	Self-Reported	Self-Reported Physician-Diagnosed	Gluten-Free Diet (Yes/No)
Celiac disease *	45 (−)	1 (1/0)	0	1	1/0
Wheat allergy	30.5 (18–45)	9 (6/3)	9 ^#^	0	3/6
NCGS **	37.2 (21–56)	12 (9/3)	2	10	2/10

* Diagnosed by gastroenterologist; ** Diagnosed by gastroenterologists (five cases) and general practitioners (five cases); ^#^ All cases were identified by means of self-reported convincing symptoms of acute allergic reactions.

Among those who reported recurrent adverse reactions to gluten (*n* = 96), we found 18 cases of self-reported gluten-related disorders (18.75%). In this group, eight respondents reported NCGS (six were physician-diagnosed), but only two of these were following a gluten-free diet (Table 2). Considering that the other four self-reported NCGS cases were not following a gluten-free diet, the general prevalence rate of self-reported NCGS currently following a gluten-free diet was 0.16% (95% CI: 0.04–0.58). On one hand, the general self-reported prevalence rate of wheat allergy currently following a gluten-free diet was 0.24% (95% CI: 0.08–0.71). Excluding the three physician-diagnosed gluten-related disorder cases adhering to a gluten-free diet (Table 2), six respondents who reported recurrent adverse reactions to gluten were following a gluten-free diet without proper diagnostic work-up of gluten-related disorders (6.25%), and thirty-five respondents reported that they were avoiding gluten in their diets (36.4%). On the other hand, nineteen respondents in this group had a physician diagnosis of colitis (19.8%) and they related the condition to gluten intake, but none of them were following a gluten-free diet.

Previous studies have used self-reported prevalence rates of adherence to a gluten-free diet among individuals without celiac disease as a surrogate marker for NCGS [15]. Consequently, the data were analyzed in this context. The estimated self-reported prevalence rate of adherence to gluten-free diet was 3.7% (95% CI: 2.7–4.8) (Table 3). Excluding the self-reported physician-diagnosed celiac disease case and three self-reported wheat allergy cases following a gluten-free diet (Table 2), the estimated prevalence of NCGS in the studied population would be 3.34% (95% CI: 2.47–4.49).

General prevalence estimations and prevalence comparisons by gender are given in Table 3. Except for the cases of dairy intolerance, all prevalence comparisons between genders were statistically significant (*p* < 0.05). Prevalence rate of recurrent adverse reactions to foods was 11.3% lower than the prevalence rate of general adverse reactions to foods, and this was statistically significant (*p* < 0.001) (Table 3). Similarly, the prevalence rate of recurrent adverse reactions to gluten was 4.1% lower than the prevalence rate of general adverse reactions to gluten, and this was also statistically significant (*p* < 0.05) (Table 3). Overall, recurrent adverse reactions to gluten represented more than 35% of the self-reported recurrent adverse reactions to any food.

**Table 3:** Study population and prevalence estimations.

Sample Size *	Assessment	(+) Cases **	Age ^#^ (Range)	Prevalence by Gender (95% CI)	*p* Value	General Prevalence (95% CI)
1238 M ^##^ = 559 F ^##^ = 679	Adverse reactions to foods	Total = 398 M = 141 F = 257	31.3 (18–85)	M 25.2 (21.8–29.0) F 37.8 (34.3–41.6)	<0.001	32.1 (29.6–34.8)
1230 M = 554 F = 676	Recurrent adverse reactions to foods	Total = 256 M = 81 F = 175	32.0 (18–84)	M 14.6 (11.9–17.8) F 22.7 (22.6–29.3)	<0.001	20.8 (18.6–23.2)
1221 M = 555 F = 666	Food allergy	Total = 67 M = 13 F = 54	27.4 (18–57)	M 2.3 (1.2–3.9) F 8.1 (6.3–10.4)	<0.001	5.5 (4.3–6.9)
1221 M = 555 F = 666	Dairy intolerance	Total = 43 M = 18 F = 25	30.8 (18–58)	M 3.2 (2.1–5.1) F 3.7 (2.6–5.5)	NS	3.5 (2.6–4.7)
1238 M = 559 F = 679	Adverse reactions to wheat/gluten	Total = 144 M = 45 F = 99	33.5 (18–71)	M 8.0 (6.0–10.6) F14.6 (12.1–17.4)	0.001	11.9 (9.9–13.5)
1237 M = 559 F = 678	Recurrent adverse reactions to wheat/gluten	Total = 96 M = 25 F = 71	34.2 (18–63)	M 4.5 (3.0–6.5) F 10.5 (8.4–13.0)	<0.001	7.8 (6.4–9.4)
1228 M = 556 F = 672	Adherence to gluten-free diet	Total = 45 M = 13 F = 32	32.0 (19–57)	M 2.3 (1.4–4.0) F 4.8 (3.4–6.6)	0.033	3.7 (2.7–4.8)

* The population school degree ranged from elementary to postgraduate; the sample size varies among assessments due to missed questions; ** Reported cases for the assessment; ^#^ Mean age (Years); ^##^ F: Female; M: Male.

The prevalence rate of recurrent adverse reactions to gluten was 7.8% (95% CI: 6.4–9.4) (Table 3). However, only 0.73% (*n* = 9) of the studied population reported both recurrent adverse reactions to gluten and adherence to a gluten-free diet. Consequently, most respondents (2.8% out of 3.7%) who were adhering to a gluten-free diet reported neither recurrent adverse reactions to gluten nor other foods. In fact, 93.3% of the respondents who reported adherence to a gluten-free diet had no self-reported physician-diagnosis of gluten-related disorders. Stratified by age, adherence to a gluten-free diet was more common in the group of respondents aged ≥39 years old than the group aged from 18 to 38 years old (6.3% *vs.* 3.0%), and this was statistically significant (*p* < 0.05).

Based on symptoms “convincing” of acute allergic reactions, general self-reported food allergy prevalence rate was 5.5% (95% CI: 4.3–6.9) (Table 3) and more than 40 foods were reported as the triggers of symptomatic adverse reactions (material S1). In addition, prevalence rates of self-reported food allergy to individual foods were also reported (material S2).

Recurrent-food-induced gastrointestinal symptoms were reported for 90 out of 96 (93.7%) respondents from the adverse reactions to gluten group, and 140 out of 160 (87.5%) from the adverse reaction to other foods group (*p* > 0.05) (Table 4). More frequently reported gastrointestinal symptoms were bloating and abdominal pain in both groups. After odds ratio analyses, bloating and constipation were the symptoms more frequently reported by the recurrent adverse reactions to gluten group compared to the opposite group (Table 4). However, a high proportion of those who reported recurrent adverse reactions to foods different from gluten experienced bloating (59.3%) and constipation was reported for less than 35% of the respondents who reported recurrent adverse reactions to gluten (Table 4). Age when gastrointestinal or extraintestinal symptoms appeared did not differ between the groups that reported adverse reactions to gluten and adverse reactions to other foods (*p* > 0.05). Diarrhea was far more common in those cases that reported adverse reactions to other foods different from gluten (19.3% *vs.* 10.0%), but this was not statistically significant (*p* > 0.05) (Table 4). There were more cases of family history of celiac disease in the group of adverse reactions to gluten (*n* = 7; 14.9%) than the group of adverse reactions to other foods (*n* = 5; 4.9%). Although this difference was not statistically significant (*p* > 0.05), risk analysis of family history of celiac disease showed an odds ratio of 3.4 (95% CI: 1.02–11.3).

**Figure 2:**
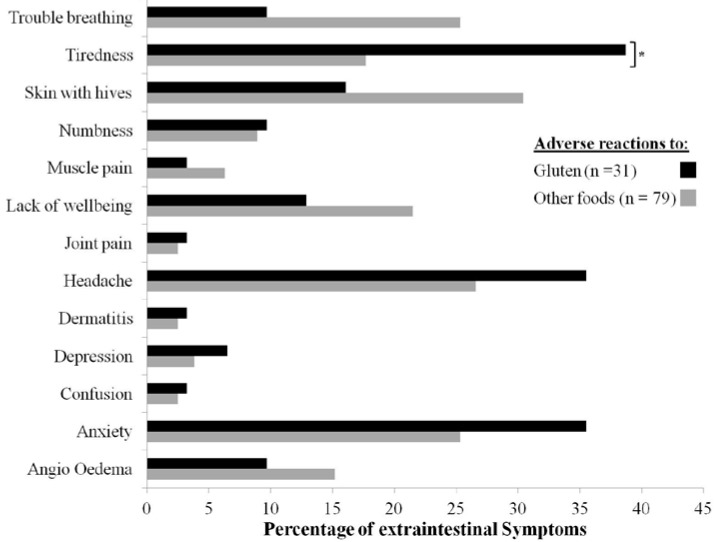
Recurrent self-reported extraintestinal symptoms. ^*^ *p* < 0.05.

Extraintestinal symptoms were less common in the recurrent adverse reactions to gluten group (*n* = 31; 32.3%) than the recurrent adverse reactions to other foods group (*n* = 79; 49.4%) (*p* < 0.05). More frequently reported extraintestinal symptoms in the recurrent adverse reactions to gluten group were tiredness (38.7%), headache (35.5%), and anxiety (35.5%) (Figure 2). In the opposite group, these symptoms were skin with hives (30.4%), headache (26.6%), trouble breathing (25.3%), and anxiety (25.3%). A statistically significant difference was found for tiredness (*p* < 0.05) this symptom being more frequent in the recurrent adverse reactions to gluten group (Figure 2). Risk analysis of this extraintestinal symptom showed an odds ratio of 2.93 (95% CI: 1.16–7.39). However, this symptom was reported for less than 40% and more than 15% of the recurrent adverse reactions to gluten group and the opposite group respectively (Figure 2). In the adverse reactions to gluten and adverse reactions to other foods groups the mean age when extraintestinal symptoms appeared was of 26.9 (Range: 5–56; *n* = 22) and 19.6 (Range: 3–50; *n* = 57) years respectively, and this was statistically significant (*p* < 0.05).

**Table 4:** Comparison between identified self-reported recurrent adverse reactions to gluten and recurrent adverse reactions to other foods cases.

Variable	Adverse Reactions to				Odds Ratio (95% CI)
	Wheat/Gluten (*N* = 90)		Other Foods (*N* = 140)		
	---	*n*	---	*n*	
Mean age in years (range) ^#^	34.1 (18–63)	---	30.5 (18-84)	---	---
Gender (female/male) (%)	73.3/26.7	66/24	63.6/36.4	89/51	1.57 (0.88–2.9)
Bloating (%)	83.3	75	59.3	83	3.4 (1.8–6.6)
Abdominal pain (%)	44.4	40	48.6	68	0.85 (0.49–1.4)
Abdominal discomfort (%)	37.8	34	30.7	43	1.4 (0.78–2.4)
Constipation (%)	34.3	31	10.0	14	4.7 (2.3–9.5)
Flatulence (%)	30.0	27	24.3	34	1.3 (0.74–2.4)
Reflux (%)	24.4	22	22.1	31	1.1 (0.61–2.1)
Acidity (%)	22.2	20	23.6	33	0.93 (0.49–1.7)
Nausea (%)	16.7	15	14.3	20	1.2 (0.58–2.5)
Diarrhea (%)	10.0	9	19.3	27	0.46 (0.21–1.0)
Vomit (%)	5.5	5	8.6	12	0.63 (0.21–1.8)
Dairy intolerance (%)	17.7	16	14.8	21	1.4 (0.72–2.9)
IBS (%)	12.5	12	8.6	12	1.5 (0.67–3.4)

^#^ Age comparison by Student t test (*p* > 0.05).

## 4. Discussion

A self-administered questionnaire for the assessment of adverse reactions to gluten and other foods was designed as a part of this study. This instrument was not reworded in its entirety, but it had to be culturally adapted to be administered in the Mexican population [26]. Previous studies have utilized questionnaires to estimate the prevalence of adverse reactions to gluten or food allergy in open populations [13,14,19,20]. However, there is not instrument designed to estimate the self-reported symptomatic adverse reactions to gluten including wheat allergy and other foods in Latin American countries. Notably, the prevalence estimation of adverse reactions to other foods different from gluten enables the measurement of the magnitude of symptomatic adverse reactions to gluten in a specific population. On one hand, the evaluation of “convincing” symptoms of food allergy not only enables the prevalence estimation of wheat allergy. On the other hand, it could also be a helpful tool to make decisions when particular cases are to be confirmed with objective diagnostic criteria. The instrument designed in this study can be reliably applied in the Mexican population and other Latin American countries after cultural adaptation. Furthermore, it measures not only variables associated to wheat allergy, but also variables associated to other gluten-related disorders.

A recent study carried out in adult Mexican population has estimated the self-reported prevalence of food hypersensitivity, defined as those adverse reactions to foods mediated or not by immunological mechanism [27,28]. Notably, no wheat hypersensitivity cases were reported. Contrary, in our study 11.9% and 7.8% of the respondents reported adverse reactions or recurrent adverse reactions to gluten respectively, highlighting that these reactions could be common in Mexican population. It should be noted that different instruments were utilized for the assessment of adverse reactions to foods, and this could partially explain the discrepancy between the two studies. Certainly, our results are in line with previous studies carried out in the United Kingdom where the estimated prevalence of gluten-sensitivity was 13% [13]. Furthermore, the estimated prevalence of self-reported wheat allergy in our study (0.72%) is in line with studies carried out in other countries where self-reported wheat allergy prevalence rates ranged from 0.5% to 1.17% [29,30,31].

A previous study carried out in Mexico reported that the prevalence of celiac disease was 0.6% (1/166) [32]. In the present study just one respondent reported a physician diagnosis of celiac disease (0.08%; 1/1238). This could be influenced in part by the fact that the frequency of classical celiac disease (symptomatic) has dropped substantially and more commonly cases are identified as non-classical celiac disease (asymptomatic) [8,33,34]. Certainly, these results suggest that celiac disease either classical or non-classical is a condition commonly underdiagnosed in the Mexican population. Therefore, screening studies of the condition in populations at risk of developing celiac disease could be helpful to avoid long-term complications.

Prevalence of NCGS largely varies among studies. Reported NCGS prevalence rates typically fluctuate between 0.55% and 18.5% [1,13,14,15]. Although most patients complaining of gluten-related symptoms did not experience symptomatic relief after gluten withdrawal [35], wheat allergy and celiac disease have to be excluded in the diagnostic work-up of NCGS [1,8]. The estimated self-reported prevalence rates of NCGS in our study ranged from 0.16% to 3.34% depending on how the analyses were conducted. Certainly, a proper diagnosis of NCGS requires not only the exclusion of other gluten-related disorders and informed symptomatic relief after gluten withdrawal, but also the gluten-attributed symptoms should be confirmed by a food challenge test [7,8]. Overall, our study highlights that prevalence estimations of NCGS should be interpreted with caution and special attention should be paid to study designs.

The self-reported prevalence of adhering to a gluten-free diet in the studied population was 3.7%. This is 6.7 fold higher than that reported in a recent study carried out in the USA where the self-reported adherence to gluten-free diet was 0.55% [15]. Unfortunately, a clear explanation of this discrepancy between studies is beyond the scope of our work. Certainly, this is probably not due to differences in the studied populations as most of the cases that reported adherence to a gluten-free diet in the USA study were over 18 years old [15]. Furthermore, in line with the USA study, we found that the prevalence of adherence to a gluten-free diet increased more than two fold among those aged ≥39 years old.

Most respondents (93.3%) who were adhering to a gluten-free diet in our study had no self-reported physician-diagnosis of gluten related disorders. Patients that benefit from a gluten-free or a wheat-free diet should be referred to a dietitian to receive dietary counseling for the diet [36]. Adhering to a gluten-free diet without proper dietary counseling could lead to iron and vitamin B deficiencies [11,12]. Furthermore, adhering to the diet without proper diagnostic work-up of celiac disease makes the practice of a six week gluten challenge in those with genetic predisposition for this condition necessary [8,37]. The main concern in this practice is that symptomatic relapse commonly precedes serology and/or histology relapse making six weeks gluten challenge intolerable for many patients [18]. Under these conditions, celiac disease could not be excluded and a proper diagnosis of NCGS would not be possible.

Previous studies have evaluated the self-reported intestinal and extraintestinal manifestations in suspected NCGS [13,14]. In this study there were evaluated the self-reported symptomatic adverse reactions to oral gluten in open population. Notably, the more commonly gluten-associated symptoms reported, either intestinal or extraintestinal, were reproducible among the studies despite different approaches or instruments were utilized to assess the manifestations. However, our results also showed that the symptoms preferentially associated with adverse reactions to gluten were not truly representative of this type of reactions as they were also frequent or unusual manifestations in those who reported recurrent adverse reactions to other foods different from gluten. These results show that symptom evaluation *per se* is not enough to confidently attribute the manifestations reported to gluten intake, and further evaluations with objective diagnostic criteria are required before making definitive conclusions.

It should be acknowledged that our study has some limitations. Firstly; this study was self-administered and questionnaire-based and the adverse reactions to foods reported, either recurrent or not, could be intoxications or other gastrointestinal conditions different from gluten-related disorders. Secondly; the use of self-reporting to estimate prevalence rates has been found to overestimate the real prevalence rates and our data were not confirmed by more specific diagnostic studies [38]. Thirdly; the instrument utilized in this study was mainly designed to estimate the prevalence of adverse reactions to gluten and consequently the manifestations evaluated were all associated to gluten-related disorders. Other variables that could be relevant to better interpretation of parts of the results were not included because of the length of the instrument. For instance, questionnaires intended to evaluate the self-reported prevalence of food allergy normally inquire about atopic diseases such as rhinitis, asthma, and eczema, and a list of potential allergens is given to improve the survey’s accuracy. Undoubtedly, the best way to evaluate prevalence of food allergy and NCGS is by designing studies that include the use of an oral food challenge, ideally double-blinded and placebo-controlled [7,8,18,39]. Thus, the main utility of self-reported questionnaire-based studies is to serve as a groundwork for further objective studies.

## 5. Conclusions

It was designed a self-administered instrument to estimate the prevalence rates of self-reported adverse reactions to gluten and other foods and adherence to a gluten-free diet. The instrument can be reliably applied in adult populations. Using this instrument, it was found that recurrent-symptomatic adverse reactions to gluten are common in Mexican population, but gluten-related disorders could be underdiagnosed. Consequently, most people adhering to a gluten-free diet had no physician-diagnosis of gluten-related disorders and this makes it highly possible that most people adhering to the diet are doing it without medical/dietary advice. To our knowledge, this is the first population-based study conducted to evaluate the prevalence of symptomatic adverse reactions to oral gluten and adherence to gluten-free diet in Latin American countries.
